# Transcriptomic analysis reveals shared gene signatures and molecular mechanisms between obesity and periodontitis

**DOI:** 10.3389/fimmu.2023.1101854

**Published:** 2023-03-29

**Authors:** Yisheng Cai, Xuemei Zuo, Yuyang Zuo, Shuang Wu, Weiwei Pang, Keqiang Ma, Qiaorong Yi, Lijun Tan, Hongwen Deng, Xiaochao Qu, Xiangding Chen

**Affiliations:** ^1^ Laboratory of Molecular and Statistical Genetics and Hunan Provincial Key Laboratory of Animal Intestinal Function and Regulation, College of Life Sciences, Hunan Normal University, Changsha, China; ^2^ Tulane Center for Biomedical Informatics and Genomics, Deming Department of Medicine, School of Medicine, Tulane University, New Orleans, LA, United States

**Keywords:** obesity, periodontitis, bioinformatics, transcriptomic analysis, immune processes, macrophage infiltration, inflammation

## Abstract

**Background:**

Both obesity (OB) and periodontitis (PD) are chronic non-communicable diseases, and numerous epidemiological studies have demonstrated the association between these two diseases. However, the molecular mechanisms that could explain the association between OB and PD are largely unclear. This study aims to investigate the common gene signatures and biological pathways in OB and PD through bioinformatics analysis of publicly available transcriptome datasets.

**Methods:**

The RNA expression profile datasets of OB (GSE104815) and PD (GSE106090) were used as training data, and GSE152991 and GSE16134 as validation data. After screening for differentially expressed genes (DEGs) shared by OB and PD, gene enrichment analysis, protein-protein interaction (PPI) network construction, GeneMANIA analysis, immune infiltration analysis and gene set enrichment analysis (GSEA) were performed. In addition, receiver operating characteristic (ROC) curves were used to assess the predictive accuracy of the hub gene. Finally, we constructed the hub gene-associated TF-miRNA-mRNA regulatory network.

**Results:**

We identified a total of 147 DEGs shared by OB and PD (38 down-regulated and 109 up-regulated). Functional analysis showed that these genes were mainly enriched in immune-related pathways such as B cell receptor signalling, leukocyte migration and cellular defence responses. 14 hub genes (FGR, MNDA, NCF2, FYB1, EVI2B, LY86, IGSF6, CTSS, CXCR4, LCK, FCN1, CXCL2, P2RY13, MMP7) showed high sensitivity and specificity in the ROC curve analysis. The results of immune infiltration analysis showed that immune cells such as macrophages, activated CD4 T cells and immune B cells were present at high infiltration levels in both OB and PD samples.The results of GeneMANIA analysis and GSEA analysis suggested that five key genes (FGR, LCK, FYB1, LY86 and P2RY13) may be strongly associated with macrophages. Finally, we constructed a TF-miRNA-mRNA regulatory network consisting of 233 transcription factors (TFs), 8 miRNAs and 14 mRNAs based on the validated information obtained from the database.

**Conclusions:**

Five key genes (FGR, LCK, FYB1, LY86, P2RY13) may be important biomarkers of OB and PD. These genes may play an important role in the pathogenesis of OB and PD by affecting macrophage activity and participating in immune regulation and inflammatory responses.

## Introduction

Obesity (OB) is a complex, multifactorial chronic inflammatory disease characterized by abnormal or excessive deposition of fat in adipose tissue (1). It is also a major risk factor for many diseases, including type 2 diabetes, cardiovascular disease, osteoarthritis and certain cancers (2). The prevalence of OB has tripled in the last few decades (3). The number of people with OB worldwide was reported to be as high as 671 million (12% of the world’s adult population) in 2016 (4). Periodontitis (PD) is one of the most common chronic multifactorial inflammatory diseases affecting the global population, leading to loss of connective tissue attachment, alveolar bone erosion, tooth loss and systemic inflammation (5, 6). There is evidence that OB increases susceptibility to PD (7). An earlier study reporting the association between OB and PD found changes in periodontal tissue in addition to greater alveolar bone resorption in obese rats compared to non-obese rats (8). Several recent studies have also suggested a comorbid effect between OB and PD (9, 10). OB increased the risk of PD by two to three times and was independent of traditional risk factors, including smoking, age, and gender (11). Animal studies have indicated that an increased alveolar bone loss in obese animals with PD and significantly greater alveolar bone loss in obese rats than in lean controls (12, 13). In addition, obese individuals who consume an excessively high-fat diet have an enhanced metabolic response to PD and show a metabolic susceptibility to increased periodontal destruction (14). These findings highlighted the existence of an association between OB and PD. However, the molecular mechanism of this association is still unknown. Therefore, exploring the common genetic features of OB and PD and their potential molecular mechanisms holds great promise for the diagnosis and treatment of OB and PD co-morbidities.

A growing body of clinical and experimental evidence suggests that immune cell infiltration and inflammatory factors play a critical role in the development of OB or PD (2, 14). On the one hand, mouse models of OB and diabetes were found to be characterized by impaired T and B lymphocyte-mediated immune responses (2). A recent study reported that natural killer T cells are regulators of adipose tissue inflammation in OB (15). Osborn O et al. (16) pointed that in the obesity-induced inflammatory response, immune cells are recruited and cause adipose tissue inflammation. Monocytes receive chemotactic signals and translocate into adipose tissue, polarizing it to a highly pro-inflammatory M1-like state. Once recruited, these M1-like macrophages secrete pro-inflammatory cytokines and act in a paracrine manner (16). In addition, OB induces a shift in the adipose tissue T-cell population, with decreased Treg content and increased CD4+ TH1 and CD8+ effector T cells that secrete pro-inflammatory cytokines (15). Recent studies have also indicated that increased B-cell numbers can promote T-cell activation and enhance M1-like macrophage polarization, inflammation, and insulin resistance (17). Notably, cytokines and chemokines from adipose tissue can also be released into the circulation and promote inflammation in other tissues in an endocrine manner (18). Meanwhile, T cells in adipose tissue are thought to play a role in obesity-induced inflammation by altering the number of adipose tissue macrophages and their activation status (19, 20). Nishimura et al. (21) showed that CD8+ T cells were increased in obese adipose tissue and promote the recruitment and activation of adipose tissue macrophages (21). On the other hand, Dutzan N et al. (22) revealed that IL-21, IL-1β, IL-17 and IL-23p19 were significantly overexpressed in periodontal disease tissues compared to healthy gingival tissues. In particular, IL-21 was overexpressed in chronic periodontitis gingival tissues and was associated with pro-inflammatory cytokines for periodontal destruction (22). IL-10 and TGF-β1 expression were down-regulated in periodontal lesions and may be regulators of inflammation and alveolar bone loss in periodontal disease (23). In addition, some researchers suggested that one of the mechanisms related to PD and OB is the increase in the production of inflammatory cytokines, i.e., OB leads to an increase in the inflammatory stimulation of adipose tissue, adipocytes secrete adipocytokines, which increases the release of inflammatory cytokines, thus leading to the imbalance between the reduction of anti-inflammatory mechanisms and persistent low-grade inflammation (24). For example, in humans, plasma levels of tumor necrosis factor α, interleukin-6 and C-reactive protein are strongly associated with OB (25). In a mouse model of OB/type 2 diabetes, resolvin E1 increased neutrophil phagocytosis in wild-type mice with Pseudomonas gingivalis, but had no effect on type 2 diabetic mice (26). Therefore, we speculate that immune factors and inflammatory responses may be one of the important reasons for the occurrence of OB and PD.

A number of previous studies have explored the potential impact of OB on the pathogenesis and progression of PD, highlighting the importance of common inflammatory processes and immune dysfunction, but the immune-related mechanisms involved in OB and PD remain to be elucidated. The aim of this study was to explore the molecular link between OB and PD using publicly available transcriptomic data. We used an integrated bioinformatics approach to study immune cell infiltration, reveal molecular regulatory networks, and explore the molecular mechanisms underlying the interaction of disease onset, hoping to provide new perspectives on the biological mechanisms of obesity-associated periodontitis.

## Materials and methods

### Data collection and processing

We searched the Gene Expression Omnibus (GEO) database for gene expression profiles of obesity and periodontitis using the keywords “periodontitis” and “obesity”. Four data sets met the inclusion criteria: (1) the experimental data type was microarray or high-throughput sequencing; (2) the number of samples per cohort was greater than six; (3) the study samples were from humans. We used the GSE104815 and GSE106090 datasets as the discovery cohort for transcriptome analysis, and the GSE152991 and GSE16134 datasets as the validation cohort. For OB, the GSE104815 dataset contained 4 OB samples and 4 non-obese samples; the GSE152991 dataset contained 34 OB samples and 11 control samples. For PD, the GSE106090 dataset contained 18 samples, of which 6 PD samples and 6 healthy samples were selected, while 6 peri-implantitis samples were excluded; the GSE16134 dataset contained 241 PD samples and 69 control samples. For the samples in these datasets, we excluded the effects of medical history and medication, as these effects may introduce bias in our study. Finally, we used the limma/DEseq2 package to filter, log2 transform, and normalize all datasets. Among these, probes were annotated as gene symbols, and for genes matching multiple probes, the probe with the highest expression value was retained. Details of the platforms, experiment types and tissues of the four datasets are shown in **Supplementary Table 1**.

### Screening for differentially expressed genes

The empirical Bayesian approach in the limma package (27) in R was applied to screen the GSE106090 and GSE104815 datasets. Significant differentially expressed genes (DEGs) were identified based on the cutoff criteria of |log2FoldChange| ≥1 and adjusted *p*-value < 0.05, and the common DEGs between OB and PD were obtained by the intersection of the plotted Venn diagram using the ggVennDiagram (28) package in R.

### Functional enrichment analysis

To further reveal the functions of the common DEGs, Gene Ontology (GO) annotation and Kyoto Encyclopedia of Genes and Genomes (KEGG) pathway enrichment analysis were performed. The biological properties of the DEGs were annotated as molecular function (MF), biological process (BP) and cellular component (CC) by GO enrichment analysis using the clusterProfiler (29) package in R, and the KEGG analysis was performed using the KOBAS (30) online tool. An adjusted *p*-value < 0.05 was set as the cut-off criterion.

### Identification of hub genes

Based on the common DEGs in PD and OB, the STRING (search tool for the retrieval of interacting genes) database (https://string-db.org/) was used to construct the PPI network, whose confidence score was set to the middle value (confidence score ≥ 0.4), and then Cytoscape software (31) was used to visualize the PPI network. The MCODE plugin (32) of Cytoscape was applied for module analysis to identify the key gene clusters, and the CytoHubba plugin (33) was used to identify the hub genes, through which four methods including maximal clique centrality (MCC), density of maximum neighborhood component (DMNC), degree and maximum neighborhood component (MNC) (34) were applied to identify the top 30 hub genes in the PPI network, respectively, and the genes obtained by taking the intersection of four gene lists were considered as hub genes.

### Validation and efficacy evaluation of hub genes

The expression matrics of GSE16134 and GSE152991 were downloaded from the GEO database and the GREIN platform (http://www.ilincs.org/apps/grein/) (35), respectively, which were used to validate the expression levels of the hub genes. GSE16134 contains 241 PD and 69 control samples, and GSE152991 contains 34 OB and 11 control samples. Considering that our data do not conform to a normal distribution, the Wilcoxon test was used to compare the two groups in the two datasets defining a *p*-value < 0.05 as significant. Meanwhile, the pROC package (36) in R was used to plot receiver operating characteristic (ROC) curves to verify the validity and predictive accuracy of the hub genes. The hub genes with an area under the curve (AUC) > 0.7 were considered useful for disease diagnosis (37).

### GeneMANIA analysis

We used the GeneMANIA online website (38) to analyze the hub genes and their co-expressed gene network by entering the validated hub genes directly into the GeneMANIA website for query, and GeneMANIA will find functionally similar genes using a wealth of genomics and proteomics data, while also reporting the weights of the predicted values for each gene used for query, and the results will show in which biological terms and pathways the genes are enriched.

### Immune infiltration analysis

Immune infiltration analysis was performed using the ssGSEA (single sample gene set enrichment analysis) algorithm (39) for GSE15299 (OB) and GSE16134 (PD), respectively. The ssGSEA algorithm is an extension of the gene set enrichment analysis (GSEA) method (40), it is a ranking based method that defines a score indicating the absolute enrichment of a specific gene set in each sample (41, 42). ssGSEA scores can be used to quantify the relative abundance of immune cells in OB or PD tissues and determine the level of immune infiltration in each sample.

The immune infiltration gene set was downloaded from http://cis.hku.hk/TISIDB/ and the immune infiltration analysis was performed using the GSVA package in R. To reveal the relationship between the hub genes and the immune cells, we then performed the correlation analysis between the hub genes and the immune cells based on Spearman’s correlation coefficients.

### Gene set enrichment analysis

To further explore the correlation between immune cell infiltration and hub genes, we performed GSEA analysis. Gene sets of pathways were obtained from the Molecular Signature Database (MSigDB) (https://www.gsea-msigdb.org/gsea/msigdb/). Based on the gene expression profile data of GSE152991 (OB) and GSE16134 (PD), the average expression value of each hub gene was calculated separately, and all samples in the dataset were divided into “high” and “low” groups according to whether the expression value of the gene was higher or lower than its average expression value, and the GSEA method was used to evaluate the relevant molecular mechanisms between the two groups. Terms with *p*-value < 0.05, |normalized enrichment score (NES)| > 1, and false positive rate (FDR) *p*-value < 0.25 were considered to be significant.

### Construction of TF-miRNA-mRNA network

Transcription factors (TFs) are proteins that can bind to specific DNA sequences and regulate gene expression (43). MicroRNAs (miRNAs) are a class of endogenous short non-coding RNAs that mediate the degradation of target mRNAs or repress translation (44). TFs and miRNAs mostly act in a combinatorial manner, where many different TFs or miRNAs control the same gene, i.e., they act synergistically on the target mRNAs (45). Moreover, TFs and miRNAs can not only co-regulate the expression of target genes but also regulate each other (46). Therefore, it is helpful to learn about the dysregulation of gene expression in various physiological and disease conditions through the transcriptional regulatory network among TFs, miRNAs, and mRNAs. The Human microRNA Disease Database (HMDD) (47) is a database for manually collecting and organizing disease-associated miRNAs, and the association information is experimentally validated. We downloaded the miRNAs associated with OB and PD from the HMDD database and obtained the co-miRNAs of OB and PD by mapping. The multiMiR package (48) provides the target genes regulated by miRNAs, which are also experimentally validated. We used the multiMiR package to find genes with possible regulatory relationships with co-miRNAs of OB and PD. The TransmiR database (49) collected TF-miRNA regulatory relationships in publications, from which co-miRNA-related TFs of OB and PD were obtained. Finally, a TF-miRNA-mRNA network was constructed and visualized in Cytoscape.

## Results

### Identification of common DEGs between OB and PD

**Figure 1:**
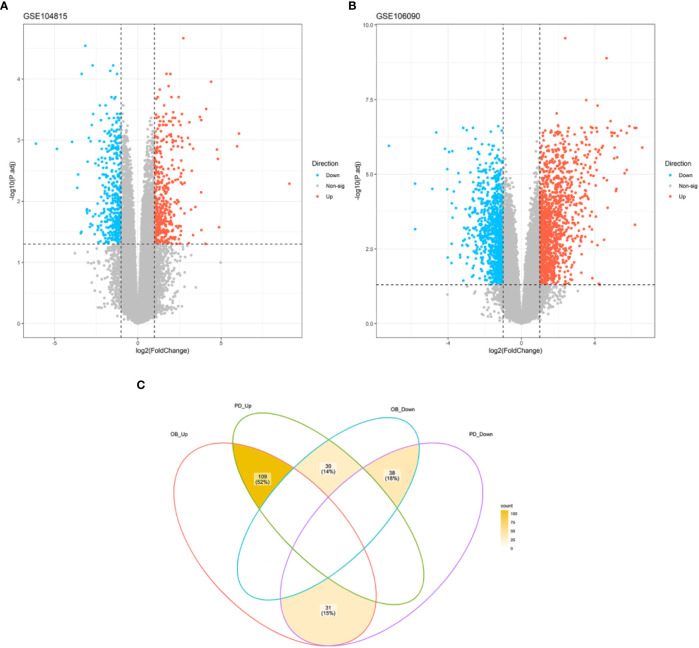
Volcano plot of DEGs and Venn diagram of common DEGs. **(A)** A total of 667 DEGs were identified between OB and healthy controls. **(B)** A total of 2191 DEGs were identified between PD and healthy controls. **(C)** A total of 109 common up-regulated genes and 38 common down-regulated genes were identified in OB and PD. DEGs, differentially expressed genes; OB, Obesity; PD, Periodontitis.

A total of 875 DEGs were identified in the OB dataset using the limma package, of which 440 genes were up-regulated and 435 genes were down-regulated (**Figure 1A**), and 2399 DEGs were obtained in the PD dataset, of which 1336 genes were up-regulated and 1063 genes were down-regulated (**Figure 1B**). After taking the intersection of the DEGs in OB and PD datasets, a total of 147 overlapping DEGs were identified, including 109 commonly up-regulated genes and 38 commonly down-regulated genes (**Figure 1C**). Heatmaps of the overlapping DEGs in OB and PD are shown in **Supplementary Figure 1**.

### GO and KEGG enrichment analysis of DEGs

**Figure 2:**
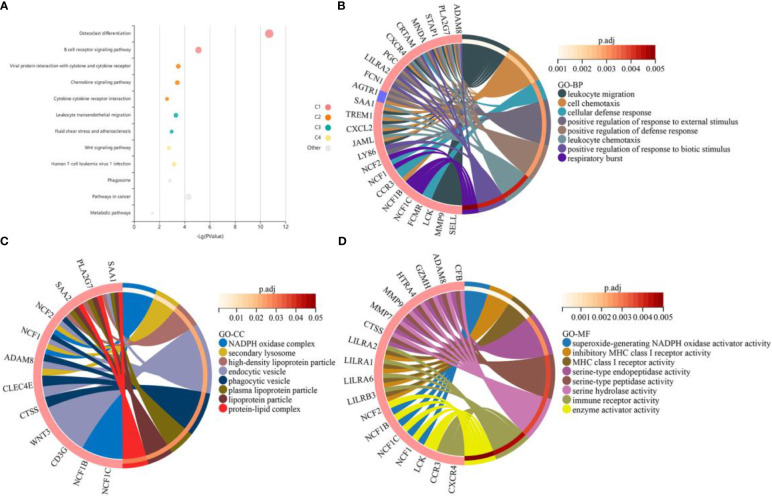
Functional enrichment analysis of the common differentially expressed genes (DEGs) between obesity and periodontitis. **(A)** KEGG pathway analysis of the DEGs. Where each bubble represents an enriched function, the size of the bubble represents 6 levels of enriched *p*-values, and the color of the bubble indicates the clustering of different pathways corresponding to different clusters (C1, C2, C3, etc.). **(B–D)** GO enrichment results of the DEGs for the categories of biological processes, cellular composition and molecular function. The red and blue colors of the gene represent up-regulation and down-regulation respectively. An adjusted *p*-value < 0.05 was considered statistically significant. DEGs, differentially expressed genes. KEGG, Kyoto Encyclopedia of Genes and Genomes. GO, Gene Ontology.

To investigate the potential biological processes and pathways of the DEGs, we separately performed GO and KEGG enrichment analysis using the clusterProfiler package and the KOBAS website. The results of KEGG enrichment analysis demonstrated that these genes were mainly enriched in “osteoclast differentiation”, “B cell receptor signaling pathway” and “viral protein interaction with cytokine and cytokine receptor” (**Figure 2A**). The results of GO analysis revealed that biological processes such as “leukocyte migration”, “cellular chemotaxis” and “cellular defense response” were significantly enriched (**Figure 2B**). In terms of cellular composition, terms such as “NADPH oxidase complex”, “secondary lysosome” and “high-density lipoprotein particle” were significantly enriched (**Figure 2C**). In terms of molecular function, terms such as “superoxide-generating NADPH oxidase activator activity”, “inhibitory MHC class I receptor activity” and “MHC class I receptor activity” were significantly enriched (**Figure 2D**). The detailed results of the GO and KEGG enrichment analysis are shown in **Supplementary Tables 2**, **3**.

### Construction of PPI network and identification of hub genes

**Figure 3:**
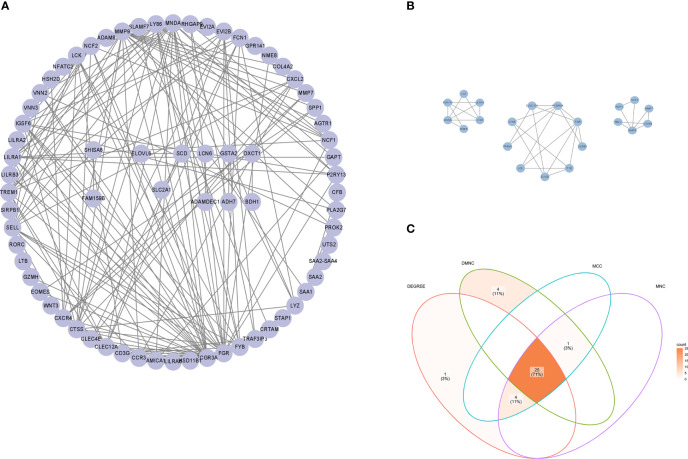
PPI network of hub genes. **(A)** PPI network of common DEGs constructed using the STRING database. **(B)** Three gene modules were identified by the MCODE plugin. **(C)** Venn diagram showing 25 common hub genes identified by MCC, MNC, Degree and DMNC algorithms using the CytoHubba plugin. PPI, protein-protein interaction; DEGs, differentially expressed genes; MCC, maximal clique centrality; DMNC, density of maximum neighborhood component; MNC, maximum neighborhood component.

To further reveal the potential relationships among the common DEGs in OB and PD, a protein-protein interaction (PPI) network of these genes was constructed in the STRING database, which contained 72 nodes and 182 edges (**Figure 3A**). Module analysis was performed using the MCODE plugin in Cytoscape to detect key clustering modules, from which three modules were obtained, and module 1 contained 9 nodes and 17 edges with a cluster score of 4.25; module 2 contained 6 nodes and 10 edges with a score of 4, and module 3 contained 6 nodes and 8 edges with a score of 3.20 (**Figure 3B**).

To explore genes that may play an important role in the co-occurrence of OB and PD, the CytoHubba plugin was used to identify hub genes. Due to the heterogeneity of biological networks, several topological analysis algorithms including MCC, MNC, Degree and DMNC were applied in our research, and the top 30 most important hub genes in the PPI network were obtained. The intersection of four hub gene lists revealed 25 hub genes: *FCGR3A, FGR, MNDA, SELL, NCF2, FYB1, EVI2B, LY86, TREM1, LILRA1, IGSF6, CTSS, CXCR4, LCK, CLEC12A, FCN1, CXCL2, VNN2 P2RY13, LYZ, CCR3, EOMES, MMP7, CD3G* and *CLEC4E* (**Figure 3C**). Details of the hub genes are shown in **Supplementary Table 4**.

### Validation of hub genes

**Figure 4:**
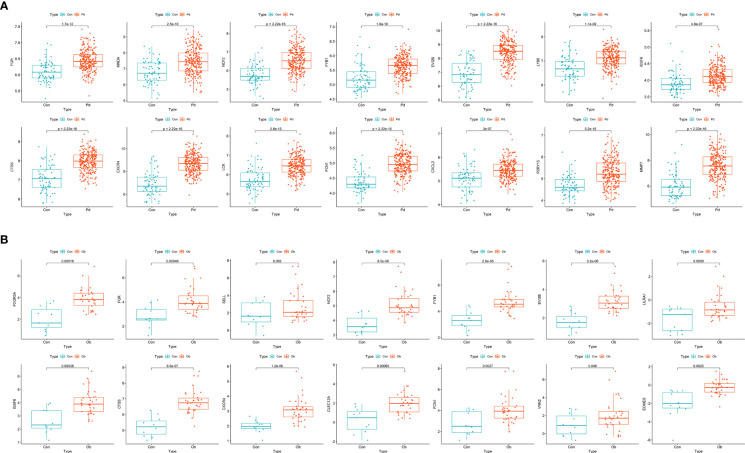
Identification and validation of hub genes. **(A)** Boxplots of the expression levels of hub genes in GSE152991. The expression levels of the 14 hub genes are significantly higher in the obesity group. **(B)** Boxplots of the expression levels of hub genes in GSE16134. The expression levels of the 14 hub genes are significantly higher in the periodontitis group. A *p*-value < 0.05 was considered statistically significant.

The 25 hub genes of OB and PD were validated using GSE152991 (for OB) and GSE16134 (for PD) datasets, respectively. The results demonstrated that 14 hub genes were significantly differentially expressed between the case and control groups in these two datasets (*p*-value < 0.05), all of which were found to be up-regulated in both OB and PD groups. The hub genes were *FGR*, *MNDA*, *NCF2*, *FYB1*, *EVI2B*, *LY86*, *IGSF6*, *CTSS*, *CXCR4*, *LCK*, *FCN1*, *CXCL2*, *P2RY13* and *MMP7* (**Figure 4**).

**Figure 5:**
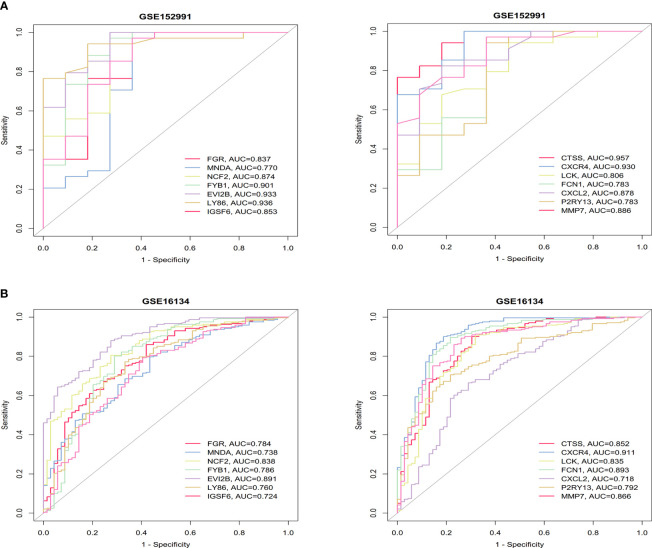
ROC curves of the 14 hub genes in obesity. **(A)** and periodontitis **(B)**. The AUC values are listed in the lower right-hand corner. ROC, receiver operating characteristic; AUC, area under the curve.

ROC analysis was performed on these two datasets to evaluate the accuracy of the diagnostic features of the hub genes. The AUC values of the 14 hub genes were all greater than 0.7 in the OB and PD datasets, indicating excellent predictive ability of these genes (**Figure 5**). The ROC curves of the hub genes in the four datasets are shown in **Supplementary Figures 2**, **3**.

### Correlation between hub genes and immune cells

**Figure 6:**
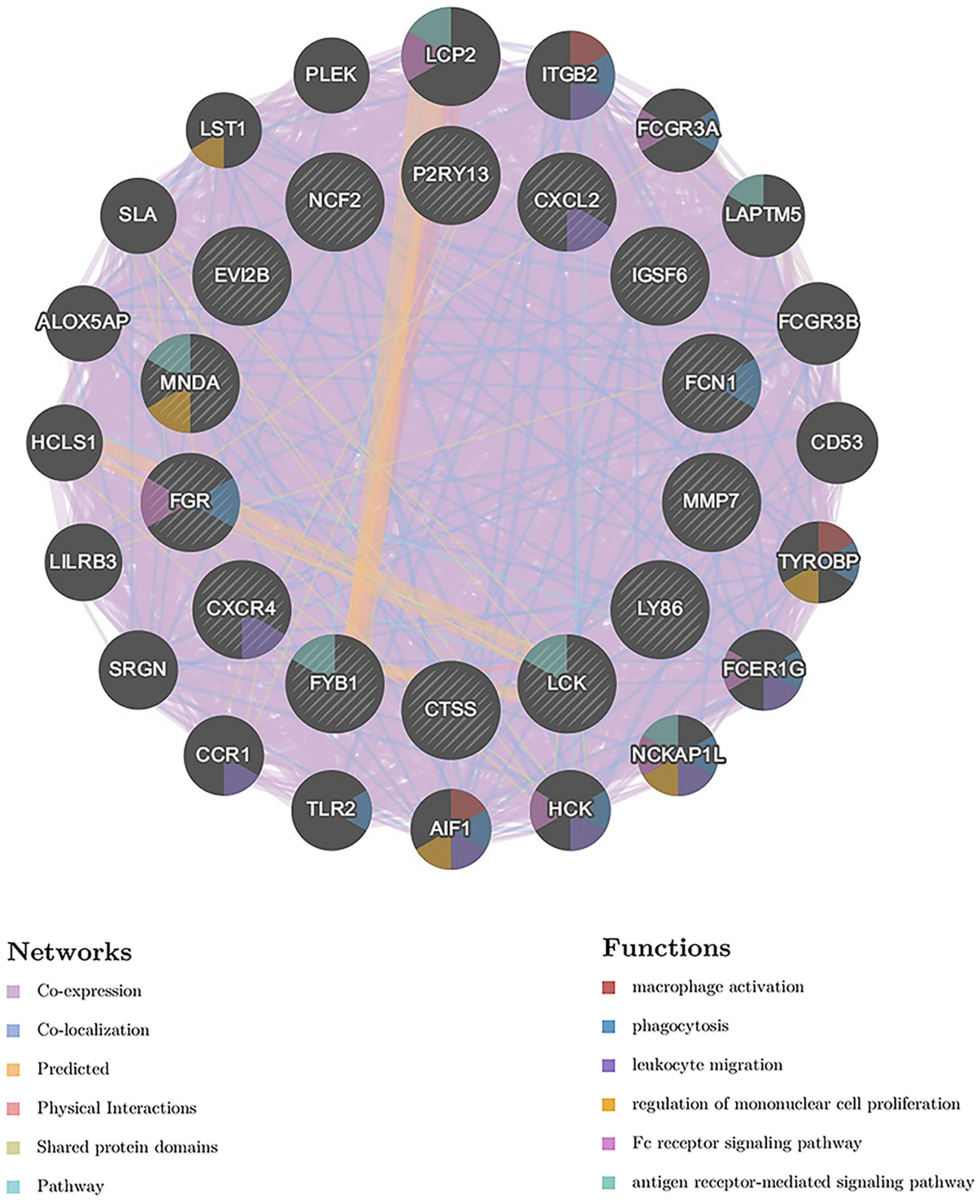
GeneMANIA analysis of the hub genes. The biological functions of the genes are shown. The hub genes are located in the inner circle and the genes correlated with the hub genes are located in the outer circle.

The 14 hub genes were imported to GeneMANIA to find correlated genes based on physical interaction, co-expression, prediction, co-localization, genetic interaction and shared protein domains. The inner circle represents the hub genes, while the outer circle represents the related genes that were newly obtained from the database. The network revealed that these genes were significantly enriched in “macrophage activation”, “phagocytosis”, “leukocyte migration”, “regulation of mononuclear cell proliferation”, “Fc receptor signaling pathway”, and “antigen receptor-mediated signaling pathway” (**Figure 6**).

**Figure 7:**
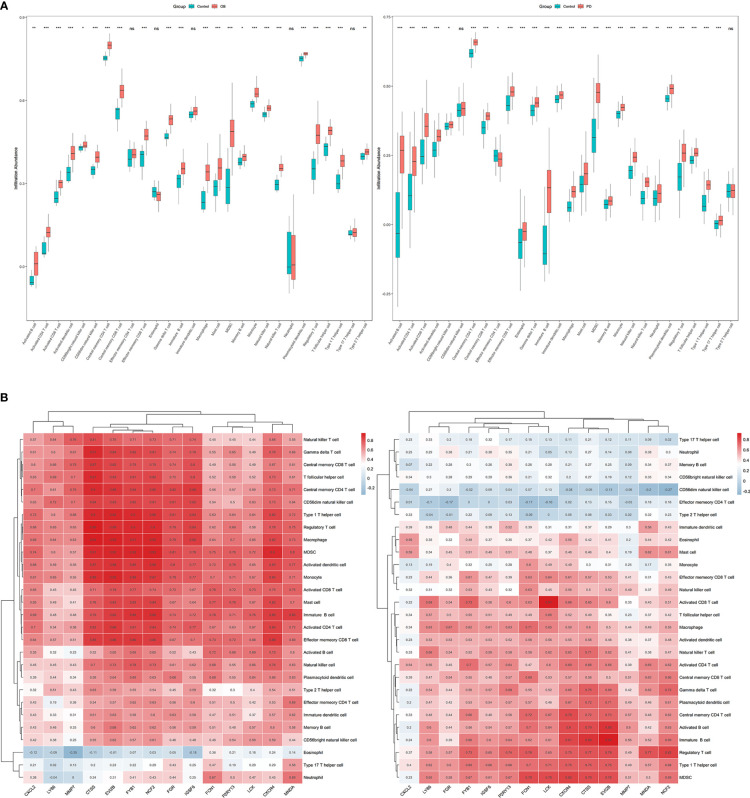
The results of immune infiltration analysis in obesity (OB) and periodontitis (PD) datasets. **(A)** Boxplots of the immune infiltration abundance in OB (left) and PD (right). **(B)** Heatmaps of the correlations between the 14 hub genes and the 28 immune cells in OB (left) and PD (right). * *p* < 0.05, ** *p* < 0.01, *** *p* < 0.001, ns, non-signicant.

Immune infiltration analysis was performed to evaluate the infiltration level of 28 immune cell types, and the correlations between the 14 hub genes and 28 immune cells were analyzed using Spearman’s method. The infiltration level of immune cells including activated CD4 T cells, activated dendritic cells, central memory CD8 T cells, immune B cells, macrophages, MDSC, natural killer T cells, and plasmacytoid dendritic cells was significantly higher in the OB and PD samples compared with the control samples (**Figure 7A**; **Supplementary Figure 4A**), and the infiltration level of immune cells such as MDSC, regulatory T cells and macrophages was positively correlated with the 14 hub genes in both OB and PD. In addition, *FGR*, *FYB1* and *LCK* were significantly associated with immature B cells, monocytes, and activated CD4 T cells. In PD samples from GSE16134, the 14 hub genes were positively associated with most cell types except effector memory CD4 T cells, type 2 T helper cells, and CD56 dim natural killer cells (**Figure 7B**; **Supplementary Figure 5**).

### GSEA results of hub genes

**Figure 8:**
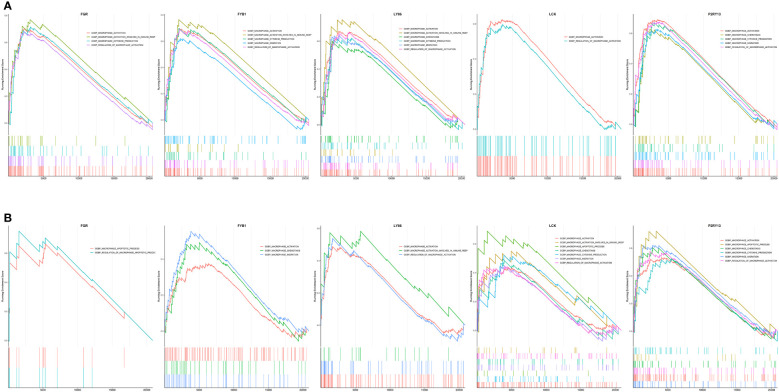
Merged enrichment plot of *FGR*, *FYB1*, *LY86*, *LCK* and *P2RY13* from gene set enrichment analysis of GSE152991 **(A)** and GSE16134 **(B)** datasets. The threshold value of GSEA results was set as |NES| > 1.0, *p*-value < 0.05 & FDR *p*-value < 0.25. GSEA, gene set enrichment analysis; NES, normalized enrichment score; FDR, false positive rate.

Both the immune infiltration results and the GeneMANIA analysis suggested that the hub genes might be closely associated with macrophages. Therefore, we explored the enrichment of hub genes in macrophage-associated pathways based on their expression in the GSE152991 (OB) and GSE16134 (PD) datasets using GSEA analysis to determine whether these hub genes are also significantly associated with macrophage-associated pathways. Macrophage-related gene sets including “GOBP_MACROPHAGE_ACTIVATION”, “GOBP_MACROPHAGE_MIGRATION”, “GOBP_MACROPHAGE_CHEMOTAXIS”, “GOBP_MACROPHAGE_ACTIVATION_INVOLVED_IN_IMMUNE_RESPONSE”, “GOBP_MACROPHAGE_ACTIVATION_IN_IMMUNE_RESPONSE”, “GOBP_MACROPHAGE_CHEMOTAXIS”, “GOBP_MACROPHAGE_CYTOKINE_PRODUCTION”, “GOBP_MACROPHAGE_APOPTOTIC_PROCESS”, “GOBP_PHAGOCYTOSIS” and “GOBP_REGULATION_OF_MACROPHAGE_ACTIVATION” were downloaded from MSigDB database and subsequently used for GSEA analysis. The GSEA results indicated that high expression of *FGR*, *FYB1*, *LY86*, *LCK* and *P2RY13* were significantly associated with several macrophage-related biological terms in both GSE152991 (**Figure 8A**) and GSE16134 (**Figure 8B**) datasets, such as “activation of macrophage”, “chemotaxis of macrophage”, “migration of macrophage”, “regulation of macrophage apoptotic process” and “macrophage activation involved in immune response”. The nominal *p*-values, NES and FDR p-values for the GSEA results of GSE152991 and GSE16134 are shown in **Supplementary Tables 8**, **9**.

### TF-miRNA-mRNA regulatory network

The information of experimentally validated miRNAs and disease associations were downloaded from the HMDD database (**Supplementary Table 5**), from which we obtained a total of 80 miRNAs associated with PD and 33 miRNAs associated with OB. And 10 common miRNA (hsa-mir-17-5p, hsa-mir-130a-3p, hsa-mir-30a-5p, hsa-mir-126-3p, hsa-mir-146a-5p, hsa-mir-21-5p, hsa-mir-24-3p, hsa-mir-155-5p, hsa-mir-200b-3p and hsa-let-7b-5p) between OB and PD were obtained.

**Figure 9:**
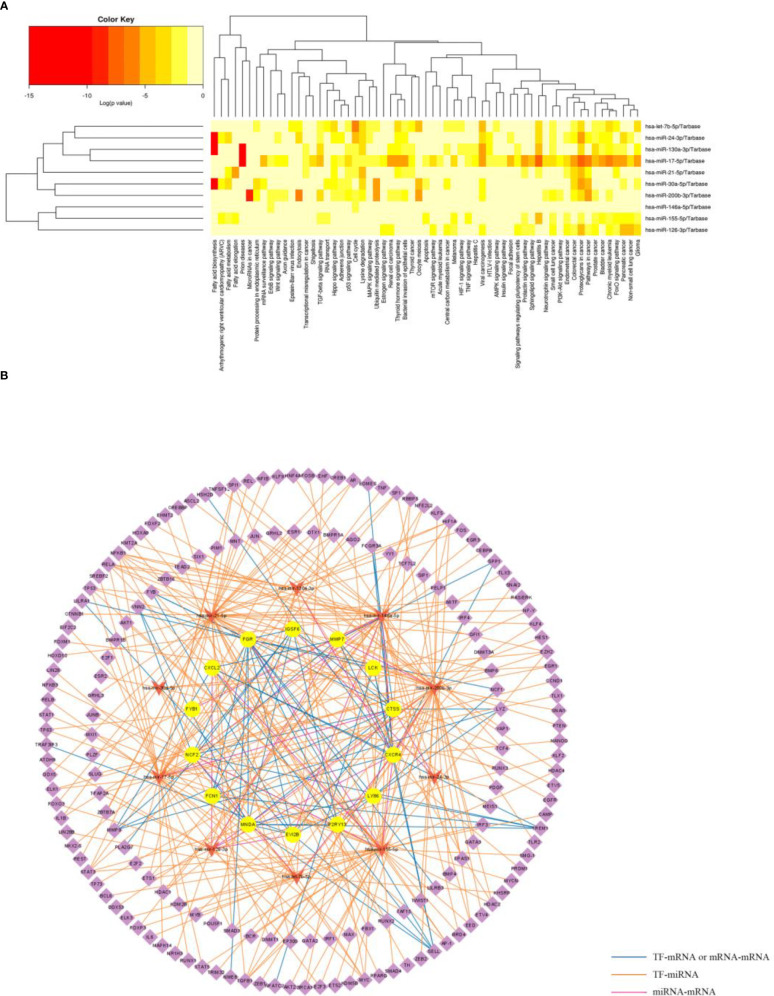
Functional enrichment analysis of the miRNAs and construction of TF-miRNA-mRNA network. **(A)** Significantly enriched biological pathways of the common miRNAs in OB and PD using the mirPath database. **(B)** The common TF-miRNA-mRNA regulatory network in OB and PD, where circles represent hub genes, inverted triangles represent miRNAs and diamonds represent transcription factors.

Functional enrichment analysis of the 10 miRNAs was then performed using the mirPath database, and the results indicated that several terms were significantly enriched, including “fatty acid biosynthesis”, “fatty acid metabolism”, “ErbB signaling pathway” and “Wnt signaling pathway and endocytosis” (**Figure 9A**). The “multiMiR” package was used to find validated target genes of the miRNAs (**Supplementary Table 6**), and the TransmiR database was used to find the regulatory information between TFs and the miRNAs, from which 233 TFs were obtained (**Supplementary Table 7**). Based on the regulatory relationships among TFs, mRNAs and miRNAs, a TF-miRNA-mRNA network was constructed (**Figure 9B**).

## Discussion

Both OB and PD are common health problems that now cause considerable economic damage and social burden worldwide (50). Previous studies have shown that the risk of periodontitis progression is 15% higher in obese than in healthy populations (51) and a systematic review of epidemiological studies has revealed a significant association between OB and PD (52). OB and PD may share overlapping pathogenic pathways, particularly immune cell infiltration and inflammation (6, 53). However, it is unclear how immune cell infiltration and inflammation accelerate the progression of OB-associated PD.

In our study, the characteristic genes shared by both OB and PD showed a close correlation with immune cell function. Based on integrated bioinformatics analysis, we screened the common DEGs between OB and PD, and functional enrichment analysis revealed that these genes were mainly involved in immune-related biological pathways such as “B cell receptor signaling pathway”, “chemokine signaling pathway”, “leukocyte migration”, “cellular defense response”, “phagocytic vesicle and immune receptor activity”. Through PPI network and hub gene analysis, we identified 25 common hub genes in OB and PD, among which 14 hub genes (*FGR, MNDA, NCF2, FYB1, EVI2B, LY86, IGSF6, CTSS, CXCR4, LCK, FCN1, CXCL2, P2RY13, and MMP7*) showed high sensitivity and specificity in the ROC curve analysis, indicating that these genes may be promising markers for diagnosis of OB and PD. GSEA analysis of the 14 genes demonstrated that 5 genes including *FGR, LCK, FYB1, LY86* and *P2RY13* were significantly involved in multiple immune-related GO terms, such as “activation of macrophage”, “chemotaxis of macrophage”, “migration of macrophage”, “regulation of macrophage apoptotic process” and “macrophage activation involved in immune response”. These results suggest that these co-DEGs may accelerate disease progression in OB and PD by affecting the activity of immune cells, especially macrophages.

Furthermore, macrophage infiltration plays a key role in inflammation in obese adipose tissue (54). In obese adipose tissue, macrophage infiltration leads to an increase in pro-inflammatory cytokines, which may lead to inflammation in other tissues *via* endocrine pathways, thus accelerating the progression of PD (55). In our study, the results of functional enrichment analysis of five common hub genes of OB and PD suggest a possible strong association with macrophages. Ortiz MA et al. (56) indicated that both *FGR* and *LCK* belong to the Src family of protein tyrosine kinases, a family of non-receptor tyrosine kinases consisting of nine members in humans: *SRC, FGR, LCK, FYN, HCK, YES, LYN, YRK* and *BLK* (56). Several Src family members are expressed in all cell types and are involved in a variety of cellular processes, of which *FGR* is mainly expressed in the hematopoietic system and *LCK* is mainly found in T lymphocytes and natural killer cells (57, 58). Like other members of the Src family, both *FGR* and *LCK* consist of a kinase structural domain, SH2, SH3 and unique N-terminal structural domains, and a regulatory C-terminal tail that phosphorylates tyrosine residues of a variety of proteins (59). *FGR* transduces signals from cell surface receptors lacking kinase activity and is involved in immunomodulatory responses, including macrophage, monocyte, neutrophil and mast cell function, cytoskeletal remodeling in response to extracellular stimuli, phagocytosis, cell adhesion and migration (60). *LCK* plays a key role in T cell antigen receptor (TCR)-related events: binding of the TCR to the peptide antigen-binding MHC complex facilitates the interaction of CD4 and CD8 with MHC class II and class I molecules, respectively, thereby recruiting the associated *LCK* protein to the vicinity of the TCR/CD3 complex. Subsequently, *LCK* phosphorylates tyrosine residues within the immunoreceptor tyrosine-based activation motif (ITAM) in the cytoplasmic tail of the TCR-γ chain and CD3 subunit, thereby initiating the TCR/CD3 signaling pathway (61). In the present study, the OB and PD enrichment results also showed that the co-DEGs were mainly enriched for molecular functions such as inhibitory MHC class I receptor activity and MHC class I receptor activity. Many studies have found that *FGR* expression is high in macrophages and monocytes and that high *FGR* expression can influence the inflammatory milieu through M1-type macrophage polarization, which in turn affects adipose tissue metabolism, leading to the development of inflammation and OB (62–64). He L et al. (65) also showed that high expression of *LCK* plays an important role in the imbalance of the immune system in periodontitis (65). In another study, *FGR* kinase was found to be a key regulator of pro-inflammatory adipose tissue macrophage activation, diet-induced OB, insulin resistance, and hepatic steatosis, knockdown of *FGR* reduced lipid accumulation and lipogenic gene expression, and low expression of *FGR* prevented macrophage polarization while preventing high-fat diet-induced OB in mice (66). This is consistent with our findings that *FGR* and *LCK* expression were significantly up-regulated in OB and PD, and functional enrichment analysis showed that they are involved in immune processes activated by macrophages. Therefore, we speculate that OB accelerates PD progression, at least in part, through immune cell infiltration and inflammatory response, and that *FGR* and *LCK* may play a key role in this process.

*FYB1*, known as adhesion and degranulation promoting linker protein (*ADAP*), is required for T cell activation as a bridging protein for the *FYN* and *LCP2* signaling cascades in T cells (67). In addition, *FYB1* can be expressed on primary natural killer cells and on lymphocyte-activated killer cells stimulated by interleukin-2, resulting in enhanced antitumor responses (68). Carmo AM et al. (69) indicated that post-translational modification of *FYB1* could lead to increased tyrosine phosphorylation by affecting T-cell receptor attachment (69). The protein encoded by the *LY86* gene is lymphocyte antigen 86, also known as MD-1 protein, a secreted glycoprotein associated with *RP105* (Toll-like receptor family protein), which plays a key role in the B-cell surface expression of *RP105*, while the *RP105*/MD-1 complex is expressed in immune cells, including B cells, macrophages and dendritic cells (70). In a genome-wide methylation analysis study of OB, the methylation level of the *LY86* gene was shown to be higher in obese cases compared to healthy controls (71). The product of the *P2RY13* gene belongs to the G protein-coupled receptor family, a 354 amino acid-encoded gastrointestinal protein-coupled receptor that is involved in the pathogenesis of purine energy transfer pathways, cholesterol metabolism, inflammation and immune dysfunction mechanisms, mediating a variety of pathophysiological processes, such as apoptosis, autophagy, proliferation and metabolism (72, 73). Many studies have shown that *P2RY13* promotes apoptosis and increases the release of pro-inflammatory factors (74, 75). In addition, *P2RY13* is highly expressed in the inflamed intestinal tissue of ulcerative colitis patients (74, 76). The above series of studies have shown that *FGR*, *LCK*, *FYB1*, *LY86*, and *P2RY13* may play an important role in the dysfunction of immune cells such as macrophages, T lymphocytes, and B lymphocytes during the pathology of OB or PD. Therefore, we speculate that macrophage infiltration and recruitment of other immune cells may be common mechanisms in OB and PD. A possible molecular mechanism is that in OB and PD, upregulation of *LY86* gene expression affects B-cell function, and the increase in B-cell number promotes T-cell activation. Meanwhile, *FYB1* is involved in T-cell activation and *LCK* phosphorylates the T-cell antigen receptor (*TCR*), but increased T cell activation may in turn promote M1-like macrophage polarization and inflammation. Among other things, *FGR* transduces signals from cell surface receptors lacking kinase activity and is involved in immunoregulatory responses such as macrophage function. Finally, *P2RY13* promotes the release of inflammatory factors.

To further explore the potential molecular regulatory mechanisms between these immune-related hub genes and immune cells, especially macrophages, we constructed a TF-miRNA-mRNA regulatory network for OB and PD. The results showed that among the five hub genes, *FGR* was regulated by hsa-miR-155-5p and *FYB1* was regulated by hsa-miR-146a-5p. In addition, TFs such as *AKT1*, *BRCA1*, and *TP63* inhibited the regulatory effect of hsa-miR-155-5p, and TFs such as *FOXP3*, *JUNB*, *NFKB1*, and *SMAD4* activated the regulation of hsa-miR-155-5p. On the other hand, the regulatory role of hsa-miR-146a-5p was inhibited by TFs such as *HDAC1*, *TP53*, and *HDAC1*, and could also be activated by TFs such as *FOXP3*, *IL1B*, and *RELA*. Notably, previous findings have shown that hsa-miR-155-5p expression is up-regulated under various inflammatory conditions (77). Langi G et al. (78) showed that the expression of hsa-miR-155-5p is down-regulated in OB patients undergoing bariatric surgery (78). In addition, miR-146a-5p expression was up-regulated in humans and mice obese adipose tissue and suppressed the inflammatory response in human adipocytes (79). Meanwhile, animal experiments revealed that deletion of the transcription factor *AKT1* increased energy expenditure and prevented diet-induced obesity in mice (80), and *AKT1* regulated macrophage polarization and alters periodontal inflammatory status (81). These findings are consistent with our study, where 14 hub genes were found to be up-regulated in both the OB and PD groups and may have a strong association with immune cells, especially macrophages. Therefore, we can reasonably speculate that the upregulation of *FGR* and *FYB1* expression in OB and PD may be regulated by hsa-miR-155-5p and hsa-miR-146a-5p and TFs such as *AKT1*, *FOXP3*, *TP53*, and *IL1B* are involved in the regulatory process of hsa-miR-155-5p and hsa-miR-146a-5p.

In this study, we used transcriptome data to elucidate the common mechanisms of OB and PD. Our study is novel in that we analyzed the infiltration of 28 immune cell species in adipose tissue of OB patients and periodontal tissue of PD patients using the ssGSEA algorithm. It is comprehensive in that we elucidated the key genes, biological pathways, immune infiltration levels, and TF-miRNA-mRNA networks of OB and PD, which will be helpful in understanding the pathophysiological mechanisms shared between the two diseases and the treatment of PD patients with OB. However, there are still limitations to our study. First of all, there is limited clinical information available in public databases, and not all datasets have large sample sizes, which may lead to bias in the results. In addition, this study was mainly based on bioinformatics analysis. Although for hub genes with high diagnostic value, we used different datasets to validate this diagnostic value, and many previous studies were able to confirm our findings to some extent, further experimental validation of our findings is still needed.

## Conclusion

Our study provides key co-diagnostic effector genes for OB and PD patients and reveals that the common key genes of both diseases are closely associated with immune cell infiltration. The possible molecular mechanism of accelerated PD progression in OB is that the secretion of pro-inflammatory cytokines increases with immune cell infiltration and inflammatory response in OB, causing inflammation in other tissues by endocrine means, thus accelerating PD progression. Five hub genes (*FGR*, *LCK*, *FYB1*, *LY86*, *P2RY13*) are promising biomarkers for OB and PD and may play an important role in the pathogenesis of OB and PD by influencing the activity of macrophages involved in immune regulation and inflammatory responses.

## Data availability statement

The datasets presented in this study can be found in online repositories. The names of the repository/repositories and accession number(s) can be found in the article/**Supplementary Material**.

## Author contributions

XQ, YC, and XC designed the study and drafted the manuscript. YZ and SW contributed to the data collection. YC, XZ, WP, and KM contributed to data analysis. YC, XZ, YZ, and SW drafted the manuscript. XQ, LT and HD revised the manuscript. All authors contributed to the article and approved the submitted version.
