# Supplementary material for: Transcriptomic analysis reveals shared gene signatures and molecular mechanisms between obesity and periodontitis
Source: Front Immunol. 2023 Mar 29;14:1101854. doi: 10.3389/fimmu.2023.1101854 (PMC10090675; doi:10.3389/fimmu.2023.1101854)
Supplement: Supplementary file 10 [file DataSheet_1.pdf]

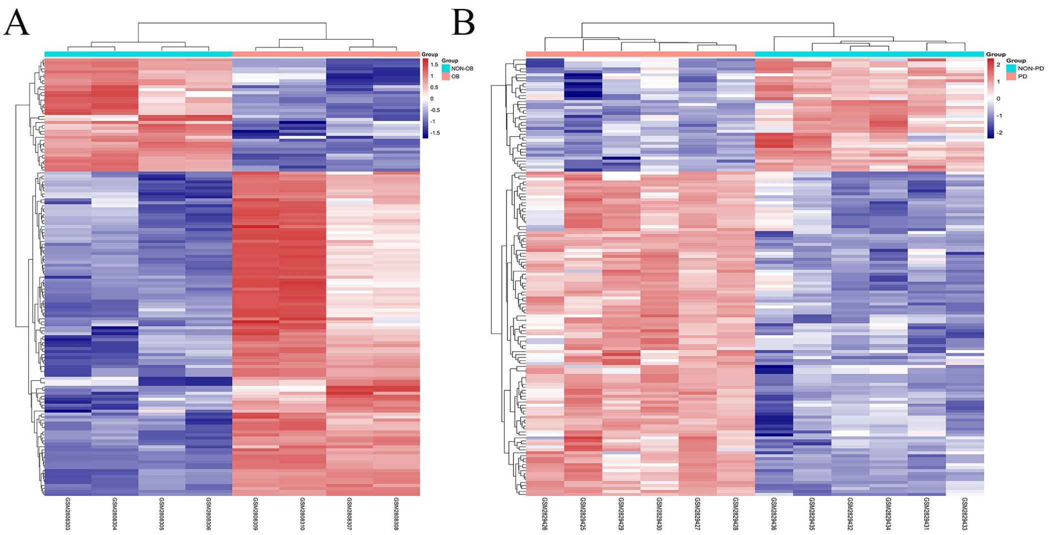

Supplementary Figure 1. Heatmaps of the common DEGs in OB (GSE104815) and PD (GSE106090).

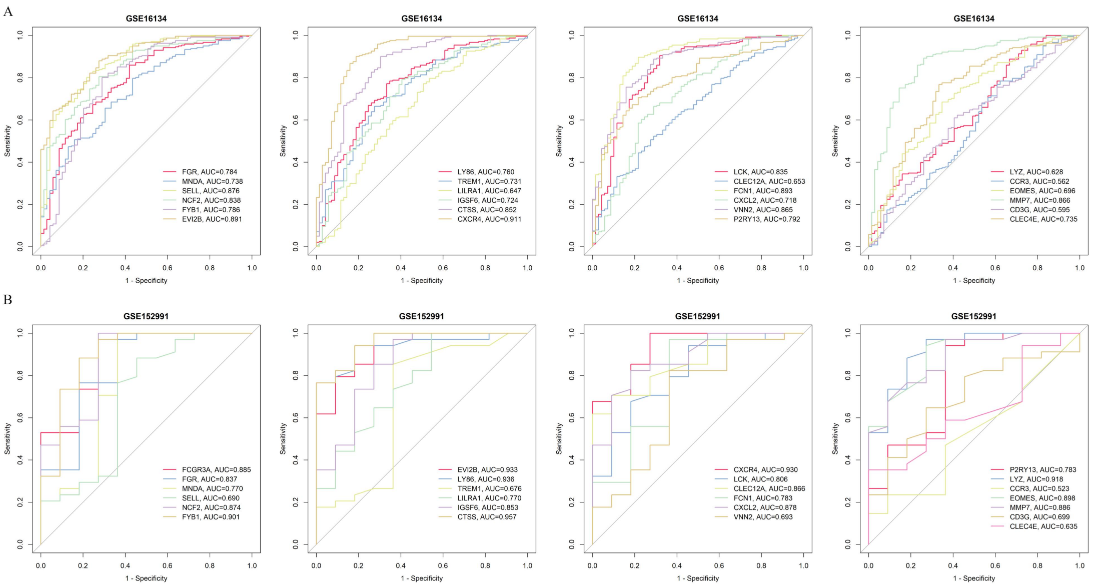

Supplementary Figure 2. ROC curves of the 25 common hub genes in GSE152991 (OB) **(A)** and GSE16134 (PD) **(B)**. The AUC values are listed in the lower right-hand corner.

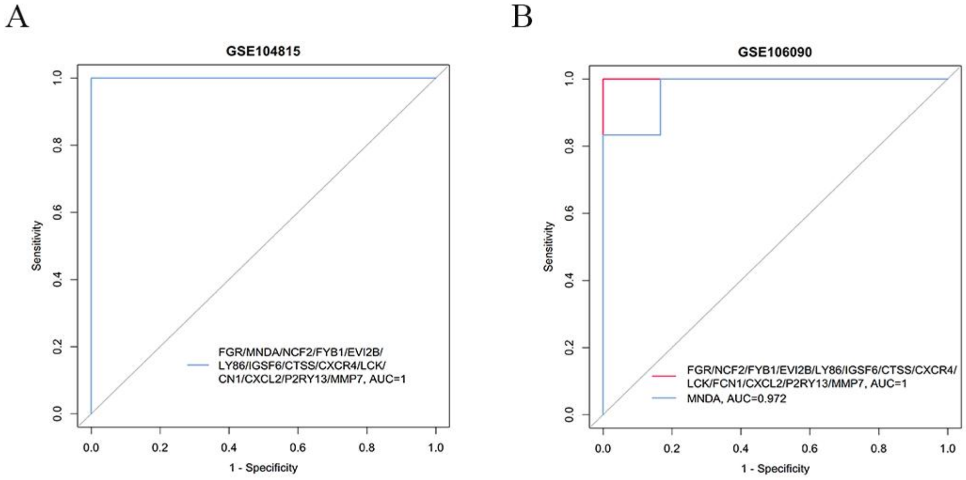

Supplementary Figure 3. ROC curves of the 14 common hub genes in GSE104815 (OB) **(A)** and GSE106090 (PD) **(B)**. The AUC values are listed in the lower right-hand corner.

A

B

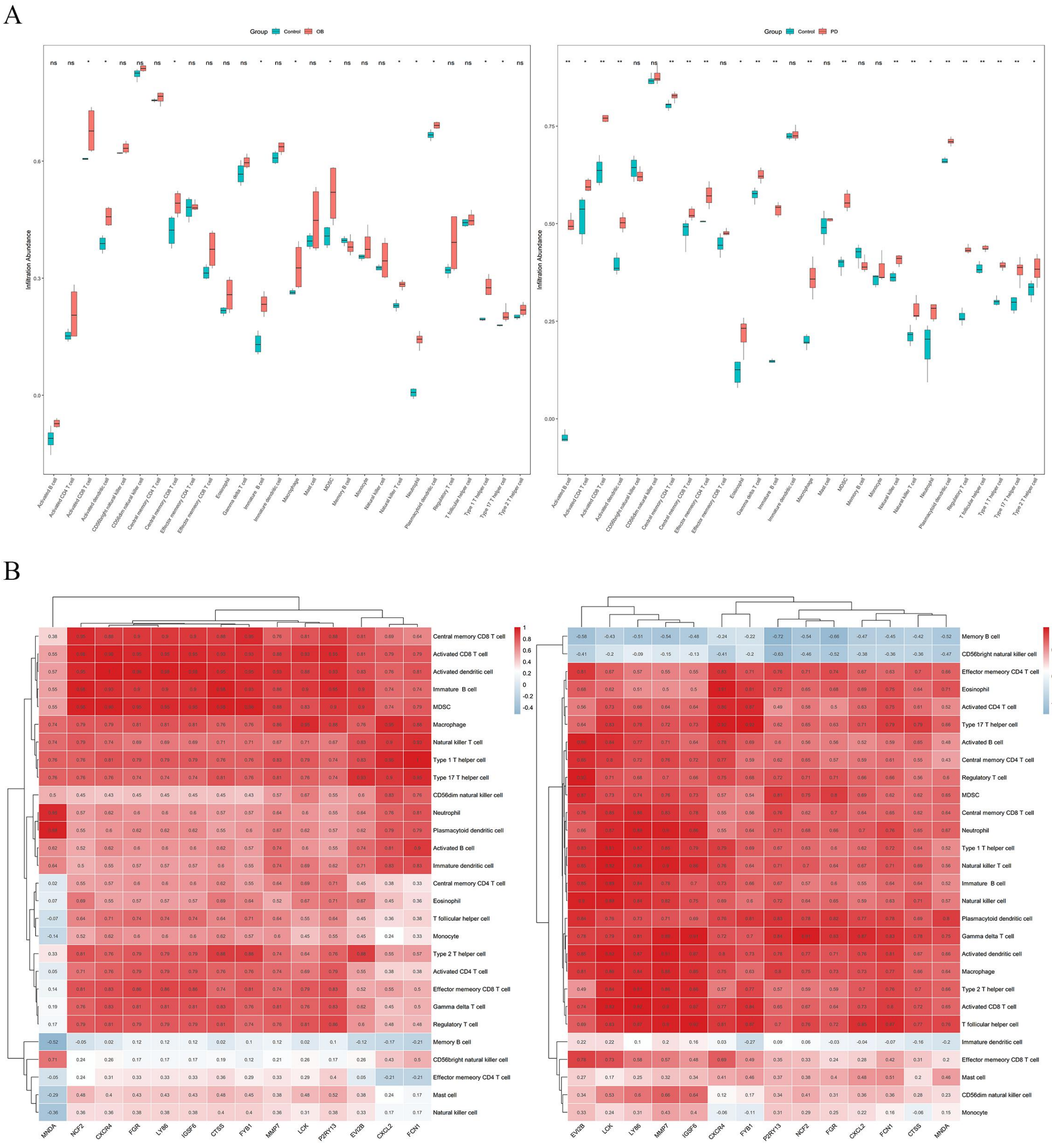

Supplementary Figure 4. The results of immune infiltration analysis in GSE104815 (OB) and GSE106090 (PD). **(A)** Boxplots of the immune infiltration abundance in OB (left) and PD (right). **(B)** Heatmaps of the correlations between the 14 hub genes and the 28 immune cells in OB (left) and PD (right). *p < 0.05, **p < 0.01, ***p < 0.001*p < 0.05, **p < 0.01, ***p < 0.001.

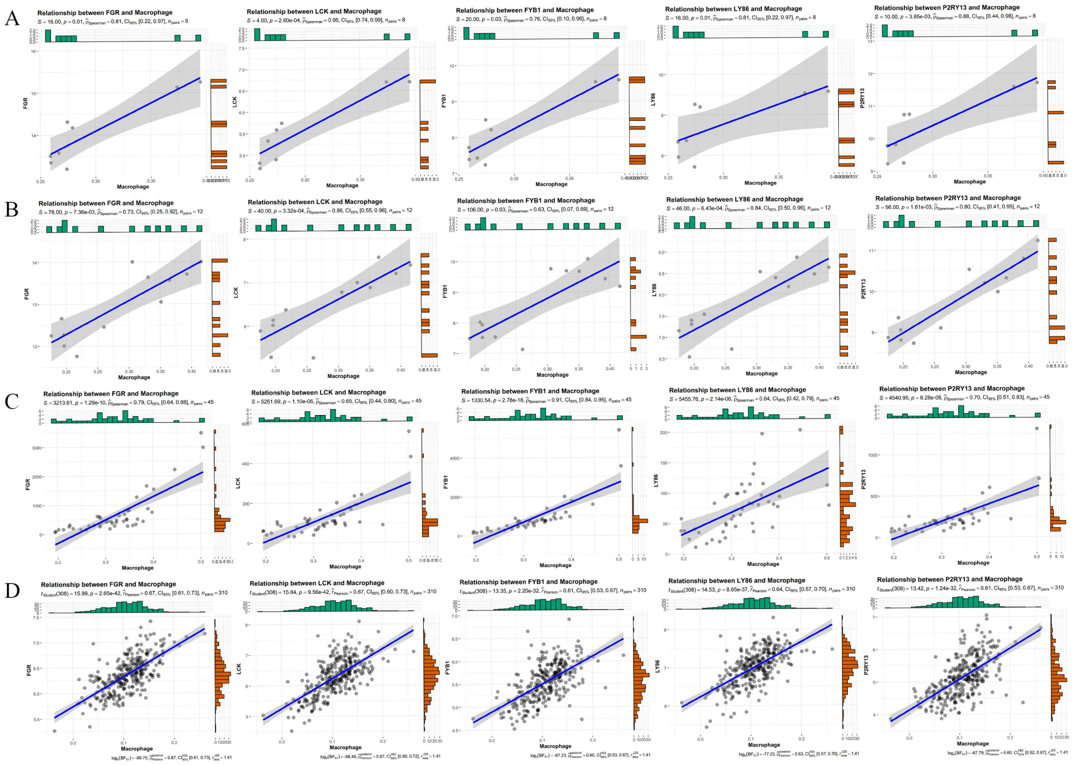

Supplementary Figure 5. Detailed correlation results of hub genes and macrophage infiltration in GSE104815, GSE106090, GSE152991 and GSE16134.
